# Iron overload accelerated lipid metabolism disorder and liver injury in rats with non-alcoholic fatty liver disease

**DOI:** 10.3389/fnut.2022.961892

**Published:** 2022-10-11

**Authors:** Lijia Zhang, Xuezheng Dai, Li Wang, Jingming Cai, Jie Shen, Yang Shen, Xianan Li, Yan Zhao

**Affiliations:** Department of Nutrition and Food Hygiene, School of Public Health, Harbin Medical University, Harbin, China

**Keywords:** NAFLD, high-fat diet, iron overload, lipid metabolism, oxidative stress

## Abstract

**Background/aims:**

Non-alcoholic fatty liver disease (NAFLD) is one of the most common liver diseases worldwide. Iron overload has been implicated in chronic non-communicable liver diseases, but its relationship with NAFLD remains unclear. This study aimed to investigate the underlying roles of iron overload in the development of NAFLD.

**Methods:**

Male Sprague Dawley rats were fed with a high-fat diet (HFD) and/or iron for 8, 12, and 20 weeks. Some rats fed with HFD plus iron also received intraperitoneal injection of deferoxamine (DFO) for 8 weeks. Liver steatosis, lipid metabolism and injury were evaluated.

**Results:**

A NAFLD model, including typical liver steatosis, was established by feeding rats with a HFD, while iron overload alone is not enough to induce severe NAFL. Compared with rats fed a HFD, excess iron further increased lipid accumulation, serum levels of lipids, enzymes of liver function, and expression levels of CD36 and FAS in rat liver. In addition, iron overload decreased the activities of antioxidative enzymes in liver compared with HFD rats. The levels of CPT1 and the ratios of p-ACC/ACC were also decreased by iron overload. DFO effectively reversed the abnormal lipid metabolism and liver damage induced by a high-fat, high-iron diet.

**Conclusion:**

A HFD plus iron overload might synergistically aggravate lipid metabolism disorders, liver injury, and oxidative damage, compared with a HFD alone. DFO might help to alleviate lipid metabolism dysfunction and improve the pathogenesis of NAFLD.

## Introduction

Non-alcoholic fatty liver disease (NAFLD) is the most common chronic liver disease in the world (1), with a global prevalence of approximately 25% (2). NAFLD encompasses a wide spectrum of liver damage, ranging from simple steatosis to non-alcoholic steatohepatitis and advanced fibrosis, ultimately leading to hepatocellular carcinoma (1, 3). Previous studies have found that patients with obesity and type 2 diabetes are more prone to liver steatosis and inflammation; in addition, patients with NAFLD often exhibit manifestations of type 2 diabetes mellitus (T2DM), such as dyslipidemia and insulin resistance (4, 5). Therefore, a better understanding of the pathogenesis of NAFLD may provide stronger evidence for the prevention and treatment of NAFLD and concomitant metabolic diseases.

A popular theory for the pathogenesis of NAFLD is the complex “multiple hit” hypothesis based on the traditional “two-hit” hypothesis (6, 7). Insulin resistance (“first strike”) promotes the entry of free fatty acids (FFAs) into hepatocytes, and then simple steatosis happens if these FFAs are unproperly metabolized or secreted. Simple steatosis leaves the liver vulnerable to second strikes, including mitochondrial dysfunction, oxidative stress, gut-derived bacterial endotoxins and inflammation (8).

Iron is an essential trace element in the human body, being a key component of hemoglobin and myoglobin, and also an essential component of cell respiration enzymes such as cytochromes, cytochrome oxidase, and catalase (CAT), which participate in crucial processes such as electron transport, redox reaction, and cell differentiation and growth (9–11). However, excess iron may initiate oxidative stress as a second “hit”, *via* the generation of reactive oxygen species (ROS) by the Fenton reaction (12). The liver is the most important organ in the body in terms of iron metabolism, and circulating iron and the iron storage of patients with NAFLD were shown to be higher than those in a control population (13). Among patients with NAFLD, the probability of iron metabolism disorders in obese children was significantly higher than that in normal children (14). In a NAFLD study, liver histological evidence indicated that more than a third of biopsy samples showed iron overload (15). This phenomenon is closely related to excessive iron deposition in the liver (16–18). Iron overload may aggravate the insulin resistance and promote the progress of non-alcoholic steatohepatitis and liver fibrosis.

It is known that excess iron can lead to oxidative injury. However, a recent study found that dietary iron overload abrogated chemically induced liver cirrhosis in rats (19). Therefore, understanding the roles of iron overload in NAFLD is extremely essential. This study aimed to clarify the effects and potential molecular mechanisms of a high-fat, high-iron diet on lipid metabolism and liver injury in NAFLD rats.

## Materials and methods

### Animals and experimental design

Six-week-old male Sprague Dawley rats (Vital River Laboratories, Beijing, China) were maintained under a 12-h light/dark cycle at 21–23°C and provided with food and water *ad libitum*. The rats were randomly divided into control, high-iron (HI), high-fat diet (HFD) (HF), HFD with low dose of iron (HFL) and HFD with high dose of iron (HFH) groups. Rats fed with HFH diet were also given an intraperitoneal injection of deferoxamine (DFO) or saline once a day from 12 to 20 weeks. The rats received the respective diets for 20 weeks. Body weight and food intake were measured and recorded weekly. Animal care and experimental procedures in this study were approved by the Animal Experimental Committee of Harbin Medical University (permission number: SCXK 2012-0001).

Rats were deeply anesthetized using sodium pentobarbital and sacrificed at weeks 8, 12, and 20, respectively. Blood, liver tissues, and epididymal, inguinal and retroperitoneal white adipose tissues were weighed and stored at –80°C for future analysis. The liver weight/body weight × 100% was calculated as the liver index. Body fat rate was calculated as the percentage of white adipose tissues to body weight.

### Dosage information

The control group was fed a 10% calorie-from-fat diet (D12450H, Research Diets). The HI group was fed a control diet plus ferrous sulfate (10 g per kilogram of diet). The HF group received a 45% calorie-from-fat diet (based on D12451 with slight modification). The rats in HFL or HFH groups were fed with a HFD plus ferrous sulfate (5 or 10 g per kilogram of diet). DFO (Novartis Pharma Stein AG, Switzerland) was given to the rats *via* intraperitoneal injection at the dose of 100 mg/kg body weight for eight consecutive weeks.

### Biochemical analysis

Serum was isolated from blood samples by centrifugation (3,000 × g, 15 min). The levels of triglyceride (TG), total cholesterol (TC), alanine aminotransferase (ALT), and aspartate aminotransferase (AST) were analyzed using an automatic biochemical analyzer (Hitachi7100, Japan). Serum ferritin levels were detected using an enzyme-linked immunosorbent assay kit (Biotopped, Beijing, China). Hepatic lipids, superoxide dismutase (SOD), glutathione peroxidase (GSH-PX), and CAT activities and malondialdehyde (MDA) contents were measured using commercial kits (Nanjing Jiancheng Bioengineering Institute, Nanjing, China) according to the manufacturer’s instructions.

### Histologic analysis

Frozen liver tissues were cut with a cryostat (Leica CM 1100), and stained with Oil Red O. Fresh hepatic tissue was fixed in 10% neutral-buffered formalin, routinely processed, embedded in paraffin, cut at 5 μm, and stained with hematoxylin and eosin (HE) for histopathological examination, and graded for steatosis (scored 0–3), hepatic ballooning (0–2) and lobular inflammation (0–3) according to the Kleiner classification criteria. The NAFLD activity score (NAS) was calculated as the sum of the steatosis, lobular inflammation, and ballooning scores, ranging from 0 to 8 (20). Sirius red staining was used to detect collagen deposition.

### Real-time reverse transcriptase polymerase chain reaction

Total RNA samples were extracted from hepatic tissues using TRIzol reagent (Invitrogen, CA, USA) according to the manufacturer’s instructions. 1μg of RNA was reverse transcribed into cDNA using a High Capacity cDNA RT Kit (ABI, USA) and real-time reverse transcriptase polymerase chain reaction (RT-PCR) was performed using an ABI 7,500 Fast real-time RT-PCR System with a Power SYBR Green Kit (Applied Biosystems, Foster City CA, USA), to detect mRNA levels of CD36, fatty acid synthase (FAS), acetyl-coA carboxylase (ACC)-1, and carnitine palmitoyltransferase (CPT)-1, with β-actin as an internal control for mRNA quantification. The primer sequences were shown in Table 1. 40 PCR cycles consisting of 15 s at 95°C and 1 min at 60°C were applied. Relative expression levels of mRNAs were calculated using the Ct (2^–ΔΔCt^) method.

**TABLE 1:** Primers used for real-time RT-PCR.

Gene	Primer sequence
CD36	Forward: 3′-GCAGCCTCCTTTCCACCTTT-5′
	Reverse: 3′-AAAGGCGTTGGCTGGAAGA-5′
FAS	Forward: 3′-TCCCAGGTCTTGCCGTGC-5′
	Reverse: 3′-GCGGATGCCTAGGATGTGTGC-5′
ACC1	Forward: 3′-AACATCCCGCACCTTCTTCTAC-5′
	Reverse: 3′-CTTCCACAAACCAGCGTCTC-5′
CPT1	Forward: 3′-TGCTGCATGGAAGATGCTTT-5′
	Reverse: 3′-CGTCGGTGGCCATGACATA-5′
β-actin	Forward: 3′-GAAGATCCTGACCGAGCGTG-5′
	Reverse: 3′-CGTACTCCTGCTTGCTGATCC-5′

### Western blot

Total protein was extracted from hepatic tissues using lysis buffer with protease inhibitors. The protein concentrations were measured with a BCA Protein Assay Kit (Beyotime Institute of Biotechnology, Shanghai, China). Twenty microgram of proteins were separated by 10% sodium dodecyl sulfate-polyacrylamide gel electrophoresis, transferred to polyvinylidene fluoride membranes (Millipore Corporation, MA, USA), incubated with 5% skimmed milk, and probed with primary antibodies diluted in blocking buffer containing 1% bovine serum albumin (BSA) at 4°C overnight. The primary antibodies used were as follows: anti-CD36 (1:1,000; Abcam, United Kingdom), anti-FAS (1:1,000; Cell Signaling, USA), anti-ACC (1:1,000; Cell Signaling), anti-phospho-ACC (p-ACC) (1:1,000; Cell Signaling), anti-CPT1 (1:6,000; Abcam), and anti-β-actin (1:800; Santa Cruz). The membranes were washed three times with TBST, incubated with the secondary antibody (1:10,000; ZSGB-Bio, Beijing, China) diluted in TBS (containing 1% BSA) for 1 h, and reacted with ECL Prime Western Blotting Detection Reagent (Beyotime Institute of Biotechnology). Signals were detected using a FluorChem-E imaging system (Protein Simple, USA). The blot signals were quantified relative to the housekeeping protein β-actin in the same sample and normalized to control. The density of the phosphorylated protein was normalized to the total protein. Triplicates were used to calculate the average density.

### Statistical analysis

All the data were presented as the mean ± SD. Differences were analyzed by ***t***-tests or one-way ANOVA followed by Student-Newman-Keuls test for multiple comparison. A ***P***-value < 0.05 was considered statistically significant.

## Results

### Iron overload aggravated lipid accumulation induced by a high-fat diet

**FIGURE 1:**
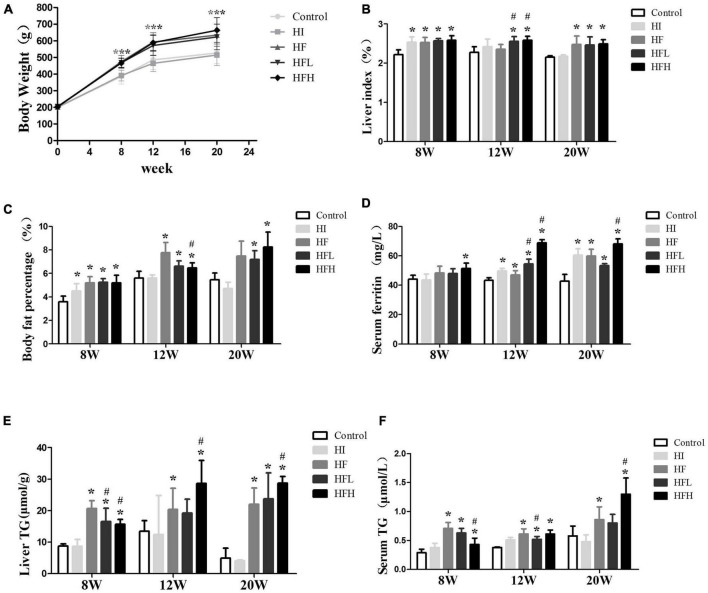
Iron overload aggravated lipid accumulation induced by a HFD. **(A)** Body weight, **(B)** liver index, **(C)** body fat percentage, **(D)** serum ferritin, **(E)** liver TG, and **(F)** serum TG levels in serum in rats at 8, 12 and 20 weeks. * and # compared with control/high-fat group at the same time point, respectively, *P* < 0.05.

Although there was no significant difference in body weights among the groups at the start of the experiment, body weight, liver index, and body fat percentage were all significantly higher in rats fed the HFD with or without iron than the control group after 8, 12, and 20 weeks. The difference between the HI and control groups was not significant for 12 and 20 weeks (Figures 1A–C).

The levels of ferritin in serum were elevated in rats fed with iron and/or HFD at 12 and 20 weeks, and high fat diet with high iron induced higher ferritin contents compared with control/high-fat group at the same time point (Figure 1D). The TG levels in the liver and serum were significantly increased in the HF and HFL groups compared with the control group at 8 and 12 weeks, and were also significantly higher in the HFH group compared with both the control and HF groups at 20 weeks although were lower compared with HF group at 8 weeks (*P* < 0.05). The average contents of liver triglyceride in HFL increased a little while in HFH were similar from 12 to 20 weeks. Serum TG levels were lower in the HFL group at 12 weeks and in the HFH group at 8 weeks compared with the HF group (Figures 1E,F).

### Iron overload aggravated hepatic steatosis and injury in non-alcoholic fatty liver disease rats induced by high-fat diet

**FIGURE 2:**
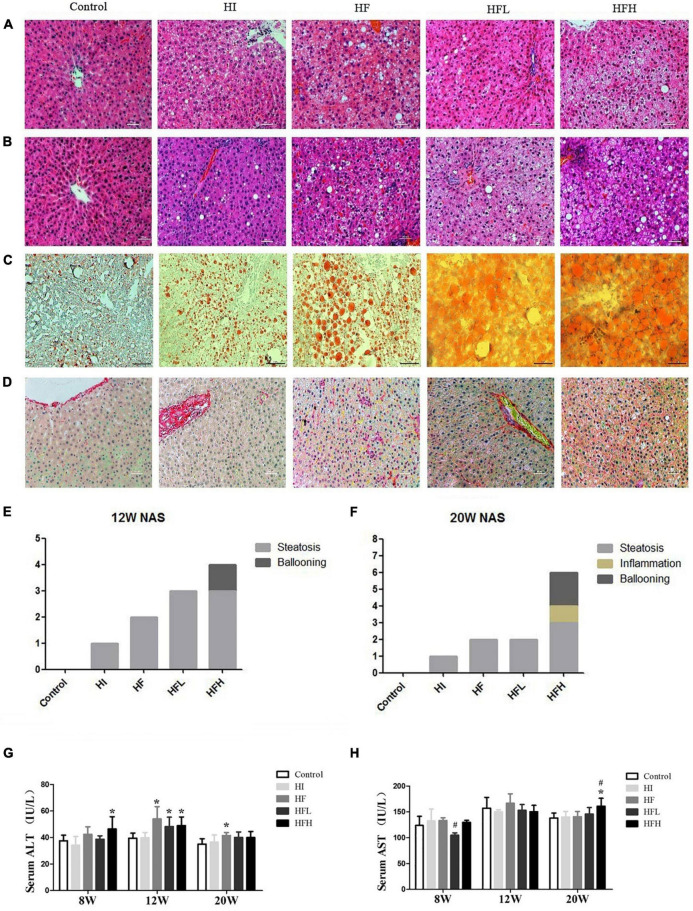
Dietary iron overload aggravated hepatic steatosis and liver injury in rats with a HFD-induced NAFLD. HE staining at **(A)** 12 and **(B)** 20 weeks. **(C)** Oil red O staining at 20 weeks. **(D)** Sirius red staining at 20 weeks (magnification × 200). NAFLD activity scores in rats at **(E)** 12 and **(F)** 20 weeks. Serum **(G)** ALT and **(H)** AST levels in rats at each time point. * and # compared with control/high-fat group at the same time point, respectively, *P* < *0.05*. Only one “* and ***” refers to the HF, HFL and HFH groups compared to the control group at the same time point, respectively, *P* < 0.05.

There was extensive lipid accumulation in the livers of rats exposed to HFD with or without iron for 12 and 20 weeks, as shown by HE staining. In addition, hepatocytes in the HFH group showed steatosis, accompanied by obvious inflammation and diffuse ballooning. Fat accounted for more than 80% of hepatocytes at 20 weeks according to Oil Red O staining (Figures 2A–C). The combined administration of high-fat and high-iron induced collagen deposition in the perisinusoidal space of rat hepatocytes, while no collagen deposition was observed *via* Sirius red staining in rats fed with HI or HF diet alone (Figure 2D). The quantified NAFLD activity scores (NAS) were consistent with the HE staining results. The volumes of lipid drops were much bigger and hepatic ballooning in HFH group was clearly visible from 12 to 20 weeks (Figures 2E,F).

ALT levels were significantly higher in the HF, HFL, and HFH groups compared with the control group at 12 weeks (***P*** < 0.05), while obvious increase of AST levels occurred at 20 weeks in HFH group compared with the control or HF groups (Figures 2G,H).

### Deferoxamine improved lipid metabolism disorder and liver injury induced by iron overload plus a high-fat diet

**FIGURE 3:**
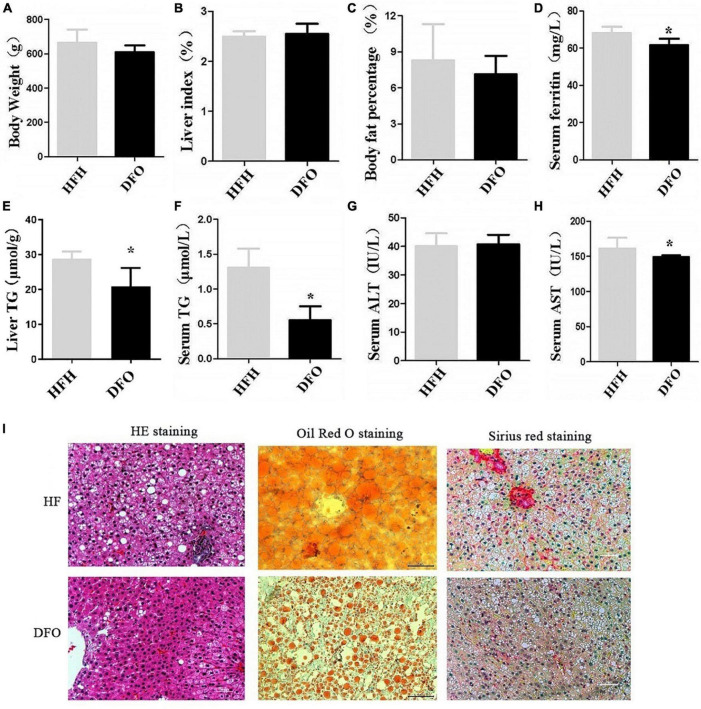
DFO treatment improved lipid metabolism and liver injury induced by iron overload plus a HFD. **(A)** Body weight, **(B)** liver index, **(C)** body fat percentage, **(D)** serum ferritin, **(E)** liver TG, **(F)** serum TG, **(G)** ALT, and **(H)** AST levels in rats. **(I)** HE, Oil red O, and Sirius red staining at 20 weeks. * Compared with the HFH group, *P* < *0.05*.

Treatment with DFO for 8 weeks had no significant effect on body weight, liver index, or body fat percentage compared with the HFH-fed rats after 20weeks (Figures 3A–C). However, DFO significantly decreased the levels of TG in both the liver and serum, serum AST and ferritin levels compared with the HFH group (***P*** < 0.05) (Figures 3D–H).

DFO treatment also reduced the amount and area of lipid droplets in the liver during the last 8 weeks of the study, as shown by HE and Oil red O staining. Collagen deposition in hepatocytes was also reduced as shown by Sirius red staining (Figure 3I).

### Iron overload plus high-fat diet impaired antioxidant capacity and induced oxidative damage

**FIGURE 4:**
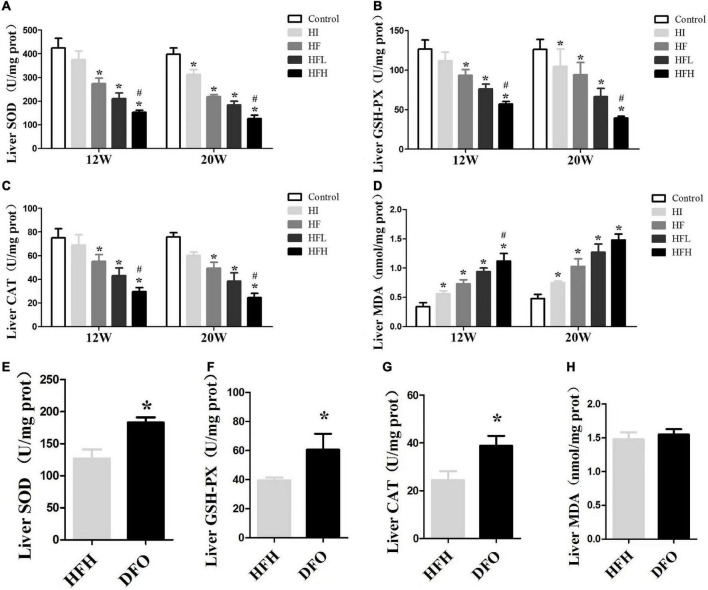
Iron overload impaired antioxidant capacity and increased oxidative damage in HFD-induced NAFLD rats while DFO attenuates this effect. **(A–D)** The activities of SOD, GSH-PX, CAT and the levels of MDA in the liver of rats. * and # compared with control/high-fat group at the same time point, respectively, *P* < 0.05. **(E–H)** The activities of SOD, GSH-PX, CAT and the levels of MDA in the liver of rats after DFO treatment. * Compared with the HFH group, *P* < 0.05.

The activities of antioxidative enzymes (CAT, SOD, GSH-Px) were significantly reduced in all groups, compared with the controls, while the levels of MDA were increased at 12 and 20 weeks. Similar changes were seen in the HFH compared with the HF group (*P* < 0.05) (Figures 4A–D). Although there was no significant difference in MDA levels between the DFO and HFH groups, DFO attenuated oxidative damage in hepatocytes of NAFLD rats induced by a high-fat, high-iron diet (*P* < 0.05) (Figures 4E–H).

### High-fat and high-iron diets affected fatty acid intake and synthesis

**FIGURE 5:**
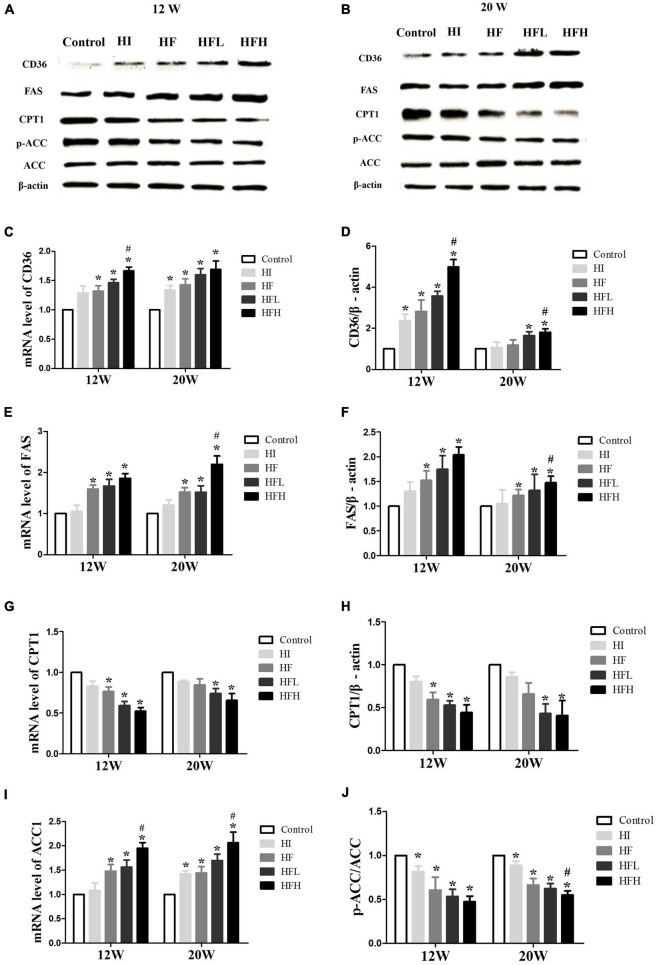
High-fat and high-iron diets affected fatty acid uptake and synthesis. **(A–J)** CD36, FAS, CPT1, and ACC mRNA and protein expression levels in the liver of rats. * and # compared with control/high-fat group at the same time point, respectively, *P* < *0.05*.

The levels of CD36 and FAS mRNA and protein were significantly higher in the HF, HFL, and HFH groups compared with the control group, while the levels of CPT1 mRNA and protein and the ratio of p-ACC/ACC protein were significantly lower when exposed to HFD with or without iron compared with the controls. In addition, CD36 and FAS expression were significantly higher in the HFH compared with the HF group (Figures 5A–J).

### Deferoxamine attenuated the effect of a high-fat, high-iron diet on lipid metabolism

**FIGURE 6:**
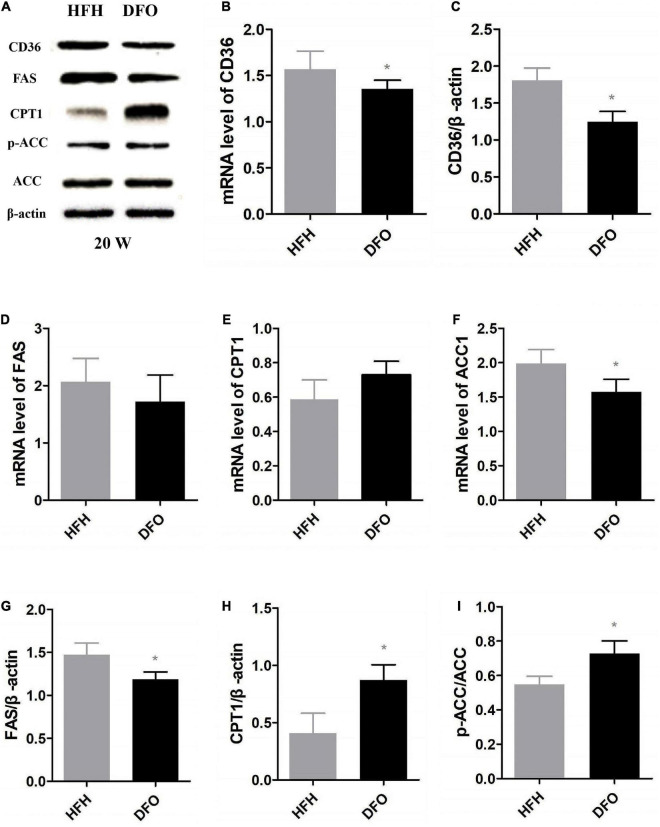
DFO attenuated the effect of a high-fat, high-iron diet on lipid metabolism. **(A–I)** CD36, FAS, CPT1 and ACC mRNA and protein expression levels in the liver after DFO treatment. * Compared with HFH group, *P* < 0.05.

CD36 and FAS mRNA and protein levels were significantly decreased after treatment with DFO, compared with the HFH group (Figures 6A–D,G). In addition, β-oxidation of fatty acids in the liver was aggravated in rats fed a high-fat, iron-rich diet. DFO also increased CPT-1 and p-ACC expression was significantly in the compared with the HFH group (Figures 6E,F,H,I).

## Discussion

Dietary administration of iron is usually applied to induce iron overload in animals, similar as genetic mutations such as hereditary hemochromatosis. Iron overload is also due to repeated blood transfusions, increased dietary iron, iron supplementation and aging. But the effects of iron overload on the liver and their underlying mechanisms as well as the origin of excess iron remain unclear. This study found that a long-term high fat diet induced steatosis, lipid accumulation, antioxidant capacity damage and lipid metabolism disorders; whereas, dietary iron overload aggravated the abnormal lipid metabolism and liver injury in NAFLD rats, and iron removal therapy by DFO efficiently attenuated this phenomenon.

Ferritin is an iron storage protein and its concentration in the serum reflects iron stores; elevated ferritin reflects risk of iron overload. In our study, the levels of serum ferritin were increased in the presence of high fat diet and/or iron at 12 and 20 weeks. Especially, high fat diet with high iron induced higher ferritin contents compared with control/high-fat group at the same time point, and DFO decreased the ferritin levels, indicating the occurrence of iron overload induced by high fat or high iron diets, and even more iron overload by synergistic administration of high fat and high iron diet.

The roles of iron in the metabolic diseases including NAFLD were still controversial (21). A series of recent studies showed that hepatic iron overload contributed to the progression of NAFLD. In NAFLD patients, excessive nutrition leads to endoplasmic reticulum stress in hepatocytes and the acceleration of intracellular iron deposition (22). However, Atarashi et al. showed that dietary iron overload could also improve chemical-induced hepatic cirrhosis (19). According to the results from the present study, HFD induced extensive lipid accumulation in the hepatocytes. The average contents of liver triglyceride in HFL were a little higher at 20 weeks than those at 12 weeks, while in HFH were similar at 12 and 20 weeks, implicating serious hepatic steatosis lasted for a long time. Liver TG levels were also significantly higher in the HFH group compared with both the control and HF groups at 20 weeks. Hepatic ballooning in HFH was clearly visible from 12 to 20 weeks. Therefore, the hepatocytes were easy to rupture and lipid droplets were dispersed when sliced. From this point of view, it was demonstrated that long-term excess dietary iron could lead to acceleration of hepatic steatosis and injury caused by HFD, supporting most recent findings. Additionally, we found that the administration of iron at low dose for 12 weeks and at high dose for 8 weeks obviously reduced HFD-triggered elevation of TG levels, in accordance with the recognition that appropriate iron was beneficial to the health. But iron overload could lead to oxidative stress and liver damage, a risk factor for the onset and progression of NAFLD (23).

**FIGURE 7:**
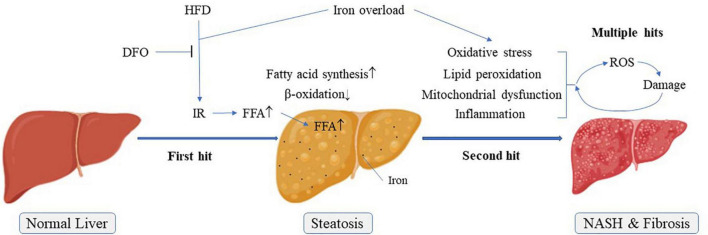
Possible pathogenesis of NAFLD “multiple hit theory”. IR, Insulin resistance; NASH, Non-alcoholic steatohepatitis.

Oxidative stress is defined as an imbalance between the production of free radicals and the antioxidant system responsible for maintaining homeostasis in the organism. Oxidative stress can lead to damage by the activation of inflammation, the secretion of proteases and the production of large amounts of oxidative products (24). Antioxidant enzyme systems, including SOD, CAT and GSH-Px, catalyze reactions to neutralize free radicals and ROS. MDA is the end-product of the peroxidation between free radicals and lipids, and can directly reflect the degree of lipid oxidation. In the present study, a combined high-fat, high-iron diet weakened the antioxidant capacity of the liver tissues and increased the MDA contents compared with a HFD alone. Notably, lipid peroxidation and antioxidant capacity had been shown to be raised or reduced, respectively, in many metabolic diseases, such as NAFLD (25). Oxidative stress and lipid peroxidation were associated with the development of NAFLD (26). Iron is an important promoter of ROS production that can initiate or catalyze the Fenton reaction and produce free radicals to destroy liver cells. At the same time, ROS produced by excess iron will further aggravate lipid peroxidation and oxidative damage (Figure 7) (27, 28).

Lipid metabolism is an important part of the pathogenesis of NAFLD. CD36 is a cell membrane transporter that plays an important role in promoting the absorption of FFAs into muscle and adipose tissue (29). In the current study, CD36 levels of mRNA and protein in the liver was significantly increased in rats fed a HFD. Previous studies showed that a HFD-induced increase in the expression of CD36 might contribute to the uptake of FFAs and the accumulation of TG in the liver (30). Expression levels of CD36 in the liver were also significantly higher in rats fed with high-fat, high-iron diets compared with those fed high-fat alone, and levels were significantly decreased after iron isolation treatment. These results suggested that long-term iron overload might promote the uptake of FFAs in the liver.

Energy produced by β-oxidation of fatty acids is an important source of energy. CPT1 is a rate-limiting enzyme for fatty acid β-oxidation and occurs in three forms in the body, of which only CPT1a is expressed in the liver (31). ACC and FAS are related to fatty acid synthesis. The present results showed that CPT1 expression levels were significantly decreased and FAS levels were increased in livers of rats fed a HFD. Moreover, the ratio of p-ACC/ACC was significantly reduced. These effects were exacerbated in rats fed with high-fat and high-iron diets. During the *de novo* fatty acid synthesis, malonyl coenzyme A could inhibit the activity of CPT1 *via* the catalyzation by ACC, a central enzyme involved in fatty acid β-oxidation and inactivated on phosphorylation (32). Inhibition of ACC phosphorylation by high fat and high iron diet could increase ACC activities, leading to the subsequent lipogenesis and accumulation. On the basis of “two-hit” hypothesis, iron overload as a multiple hit might aggravate lipid peroxidation and oxidative stress, leading to the release of inflammatory factors. All these formed multiple hits to promote the progress of non-alcoholic steatohepatitis and liver fibrosis. Interestingly, iron chelation reversed the levels of these lipid metabolism-related genes and proteins, implicating that long-term iron overload might promote the synthesis of FFAs, whereas inhibit the consumption of FFAs in the liver, leading to more serious lipid metabolism disorder than NAFLD itself (Figure 7).

Several studies also showed that hepatic iron deposition could play a role in the pathogenesis of NAFLD (33–35). In our study, both iron overload and high fat induced liver steatosis in rats, and their synergistic effects further aggravated this damage with obvious inflammation and fibrosis (Figure 7). DFO, an iron chelator, has been proved to be helpful for the protection of nerves and diabetic wound healing (36–38), DFO can also have a beneficial effect on improving adiposity by inhibiting oxidative stress and inflammation (39). The use of DFO significantly reversed this combined effect of iron, mainly by inhibiting the liver’s damage resulting from lipid peroxidation and oxidative stress, thereby alleviating lipid metabolism disorder in the liver caused by iron overload, not by HFD. Therefore, our findings provided powerful evidence for the involvement of iron overload in the pathogenesis of NAFLD. Meanwhile, DFO might be considered as a potential candidate for the treatment of NAFLD.

Interestingly, the iron overload in the HI group significantly increased body weight, liver index and fat content at 8 weeks, but there was no difference at 12 and 20 weeks, compared with the control group. It was supposed that iron supplementation for rats at a period of rapid growth for short time might help to function as a growth promoter. But as time went, the body could be adapted to iron supplementation and the promotion disappeared. In addition, the HI group was also overloaded with iron, but no adverse effects were observed when exposure to iron alone for 20 weeks in our study, except high levels of serum ferritin and lower antioxidant capacity. It was not paradoxical that excess iron may initiate oxidative stress as a second “hit” in the presence of lipid accumulation.

There were also some shortcomings in the present study. First, iron toxicity can influence major tissues involved in glucose and lipid metabolism and organs attacked by related complications. It was demonstrated that iron overload resulted in the disturbance of the lipid metabolism. However, there was an extensive interaction network or among various kinds of factors in the body. The elevation of iron storage might be associated with other factors or specific nutrition. Second, this study made a preliminary exploration on the roles of DFO in the treatment. DFO was applied after the accumulation of excess lipid and iron in the liver. It was worth investigating further whether DFO could be used at the beginning of the experiment for the prevention of iron overload and NAFLD or even liver damage. In summary, we demonstrated that a HFD caused NAFLD in rats, and that concurrent iron overload could further aggravate lipid metabolism disorders by promoting the transport of FFAs to the liver, the synthesis of endogenous fatty acids, and inhibiting fatty acid β-oxidation, resulting in lipid accumulation and oxidative damage in the liver during the development of NAFLD. Iron removal may help to relieve lipid metabolism dysfunction and improve NAFLD. These findings may provide new insights into the prevention and treatment of NAFLD.

## Data availability statement

The raw data supporting the conclusions of this article will be made available by the authors, without undue reservation.

## Ethics statement

This animal study was reviewed and approved by the Animal Experimental Committee of Harbin Medical University.

## Author contributions

LZ performed the experiments and wrote the manuscript. XD and LW wrote the manuscript. JC and JS performed the experiments. YS and XL contributed to review and editing. YZ conceived and designed the experiments and wrote the manuscript. All authors contributed to the article and approved the submitted version.
