# Supplementary material for: Iron overload accelerated lipid metabolism disorder and liver injury in rats with non-alcoholic fatty liver disease
Source: Front Nutr. 2022 Oct 11;9:961892. doi: 10.3389/fnut.2022.961892 (PMC9593083; doi:10.3389/fnut.2022.961892)
Supplement: Supplementary file 1 [file Presentation_1.PPTX]

## Slide 1
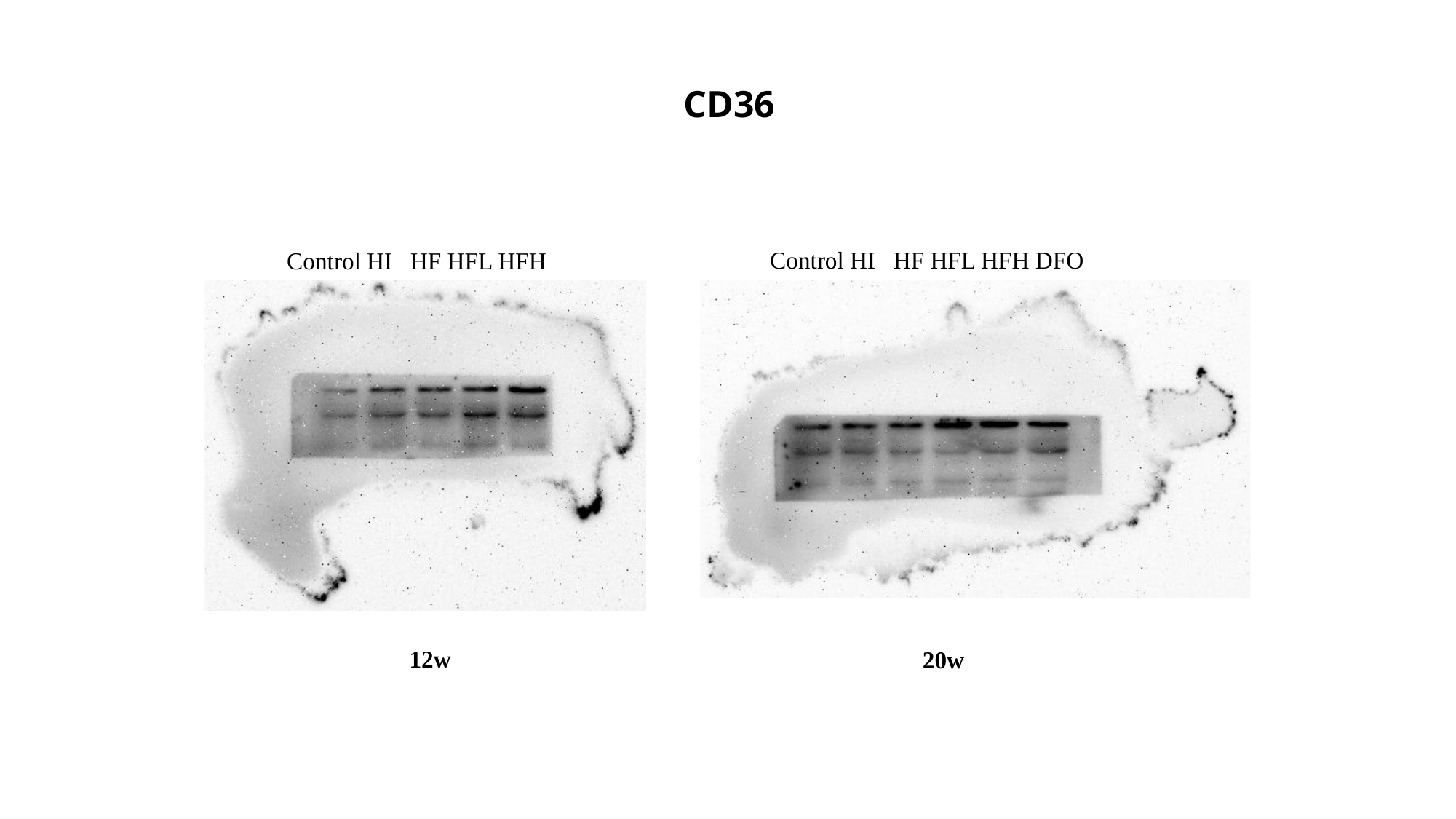

CD36
Control HI HF HFL HFH DFO
Control HI HF HFL HFH
12w
20w

## Slide 2
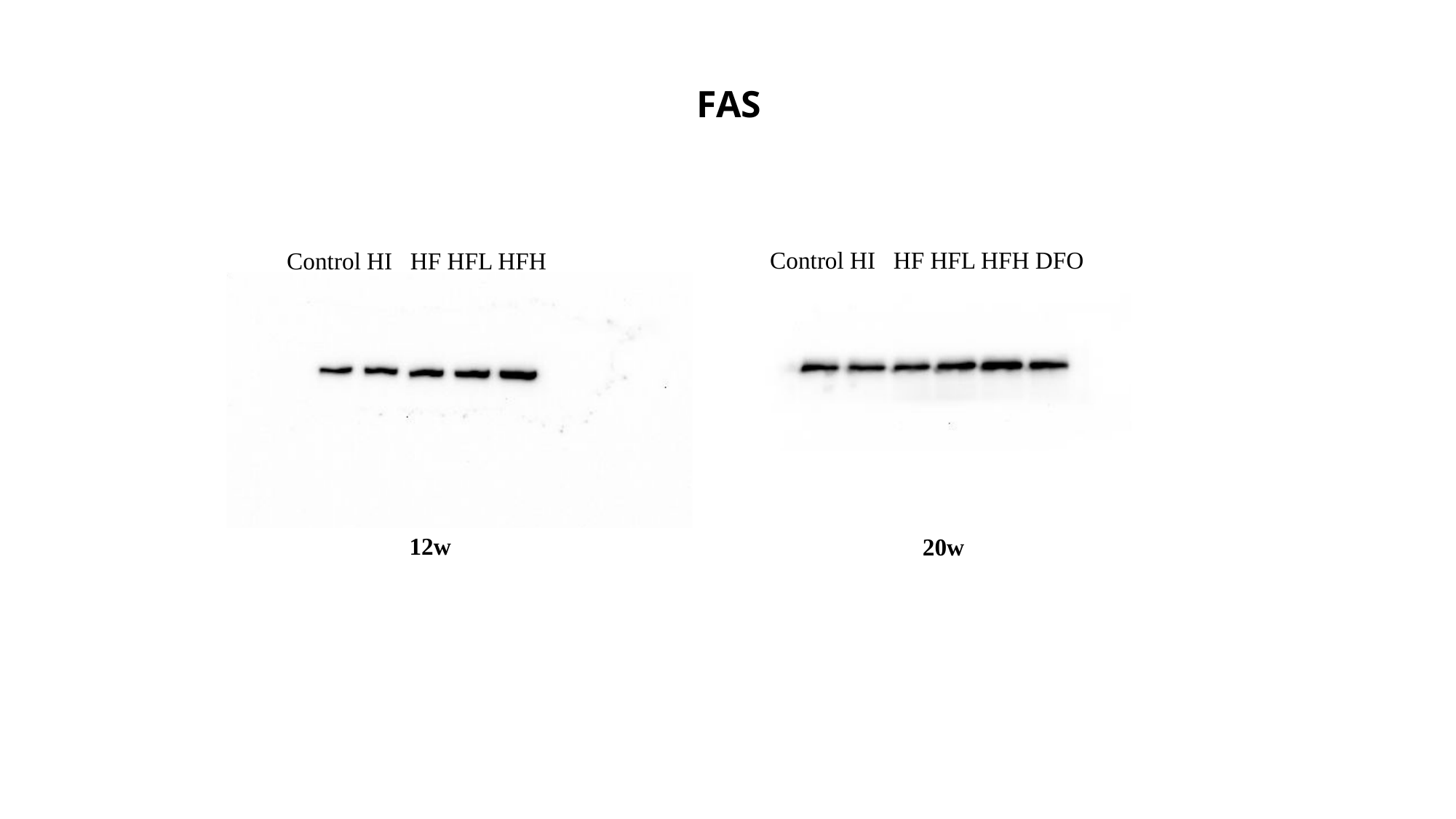

FAS
Control HI HF HFL HFH DFO
Control HI HF HFL HFH
12w
20w

## Slide 3
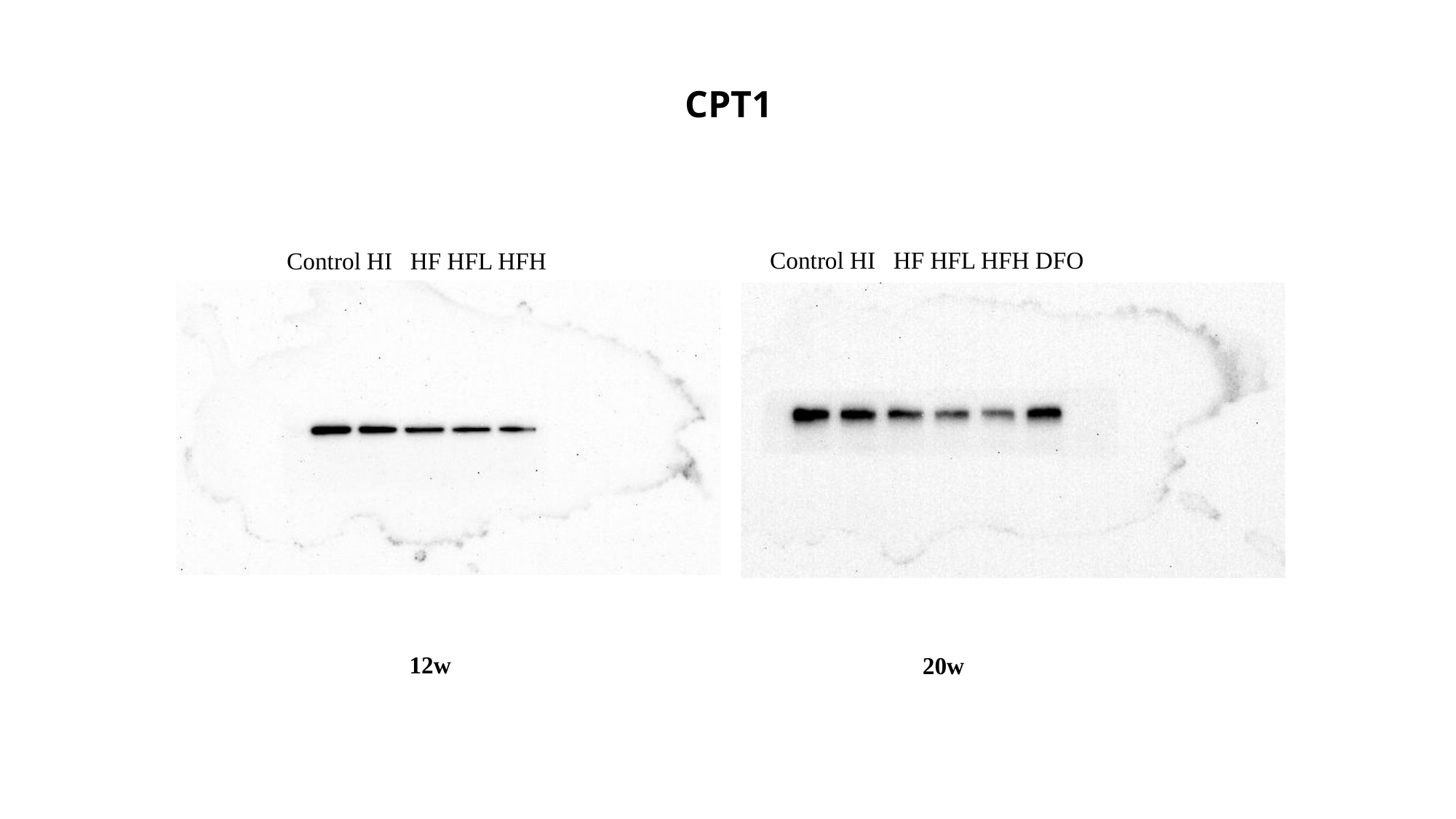

CPT1
Control HI HF HFL HFH DFO
Control HI HF HFL HFH
12w
20w

## Slide 4
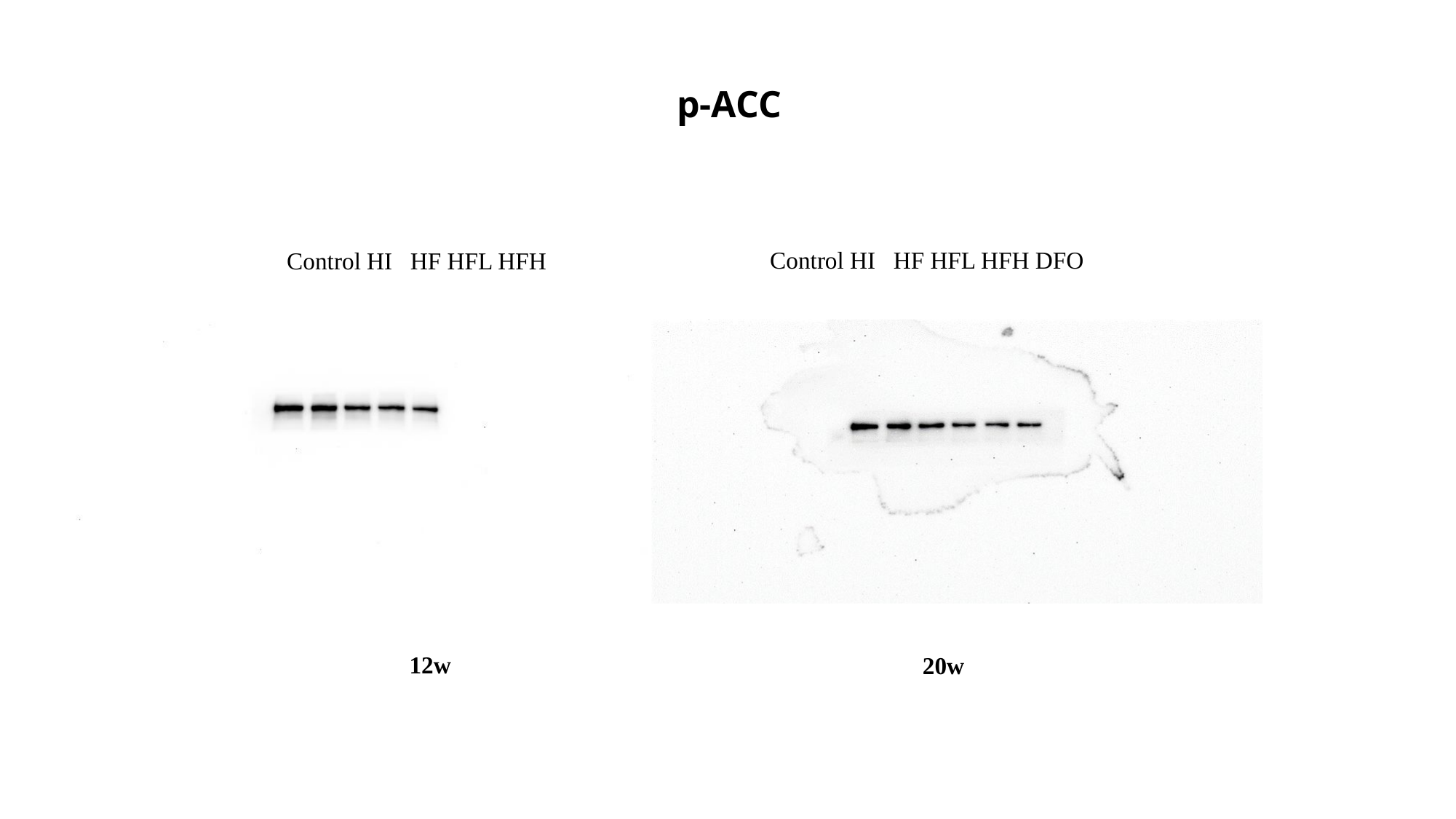

p-ACC
Control HI HF HFL HFH DFO
Control HI HF HFL HFH
12w
20w

## Slide 5
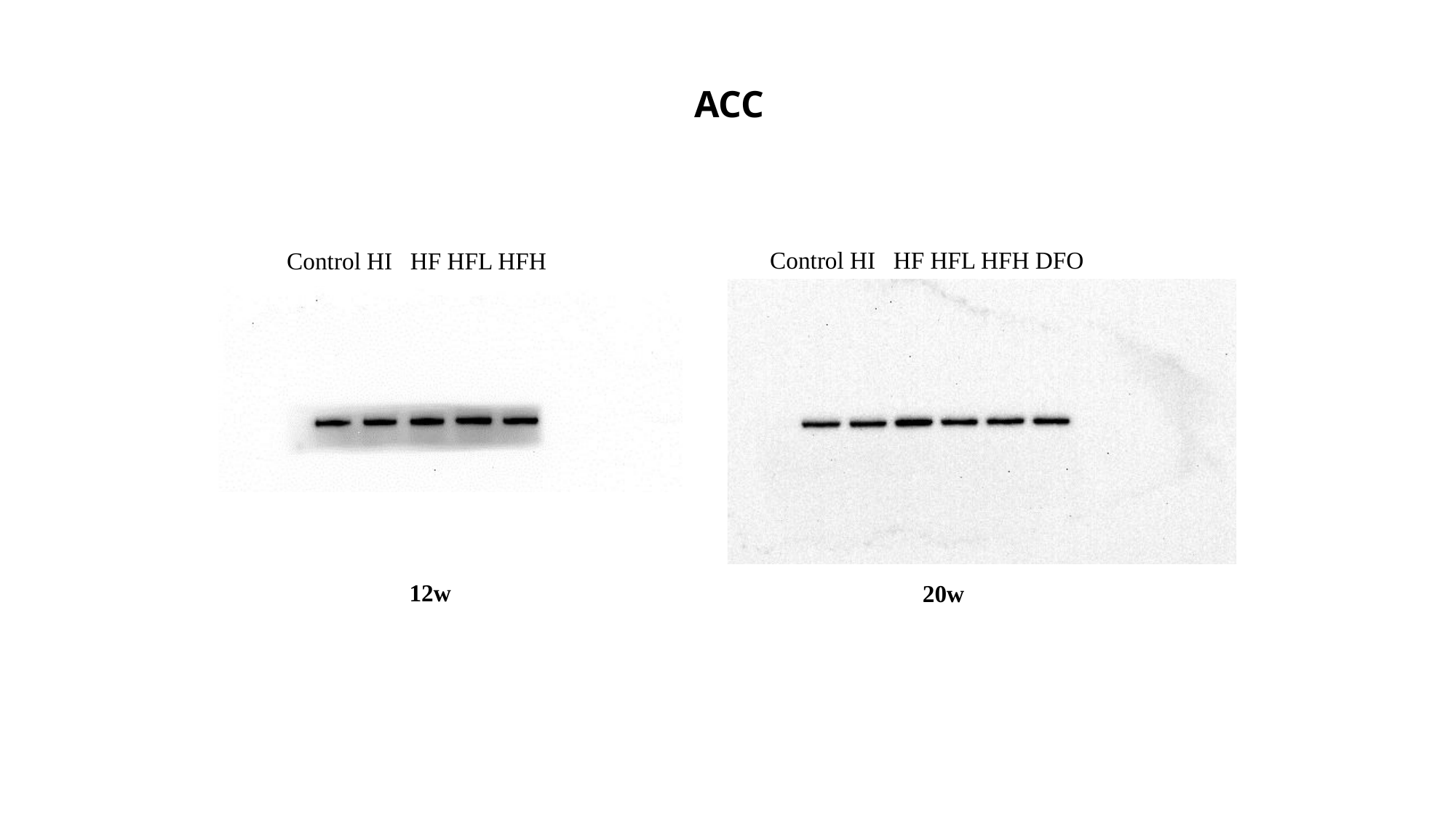

ACC
Control HI HF HFL HFH DFO
Control HI HF HFL HFH
12w
20w

## Slide 6
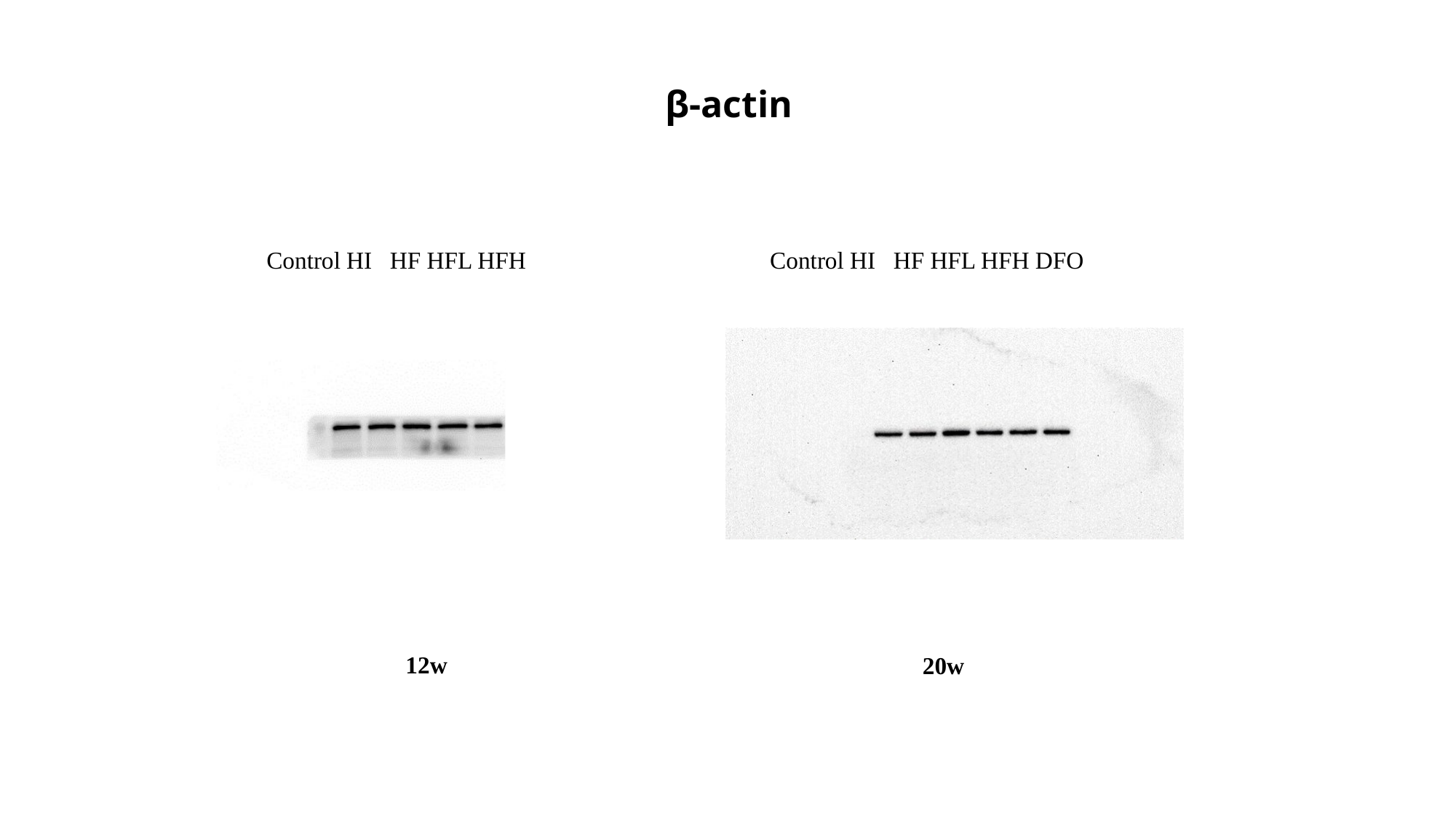

β-actin
Control HI HF HFL HFH
Control HI HF HFL HFH DFO
12w
20w
